# Oncologists’ Knowledge, Practice and Attitude toward Fertility Preservation: A National Survey

**DOI:** 10.3390/life13030801

**Published:** 2023-03-15

**Authors:** Ahmed Al Ghaithi, Eyas Al Rashdi, Maryam Al Shukri, Rahma Al Ghabshi, Halima Albalushi

**Affiliations:** 1 College of Medicine and Health Sciences, Sultan Qaboos University, Muscat 123, Oman; 2 Department of Obstetrics and Gynaecology, Sultan Qaboos University Hospital, Muscat 123, Oman; 3 Department of Obstetrics and Gynaecology, Royal Hospital 111, Muscat 123, Oman; 4 Department of Human and Clinical Anatomy, Sultan Qaboos University, Muscat 123, Oman

**Keywords:** fertility, fertility preservation, oncofertility, oncologists, cancer, care, survivors

## Abstract

**Simple Summary:**

Improved chemotherapy and radiotherapy treatment protocols fortunately increased the survival rates over the years, and more improvement is anticipated in the coming years. However, these treatments are accompanied with late effects and may result in infertility or subfertility in those survivors. Sadly, most cancer survivors realize the effects of the treatment they received on their fertility very late, when they failed attempting to have children. Thus, it is of great importance that options for fertility preservation are offered and/or made available for young cancer survivors. Therefore, health care providers should be aware of the existence of fertility-related issues in their patients and to be able to discuss the impact of the cancer and its treatment on their fertility and possible fertility preservation options. In this study, we aim to explore the perspective of oncologists and physicians dealing with and treating patients with cancer on fertility preservation. We demonstrated in this study that oncologists in Oman are supportive of fertility preservation. The lack of knowledge and unavailability of well-structured fertility preservation services in the country hinders the initiation of fertility preservation discussions. Our findings will pave the way for improving the care for cancer survivors for better quality of life.

**Abstract:**

Background: Improved chemotherapy and radiotherapy treatment protocols, fortunately, increased the rates of cancer survivors over the years. However, these treatments may result in infertility or subfertility. Oncologists are considered the gateway for knowledge about cancer and its treatments’ effects. Several studies showed that many oncologists do not discuss fertility preservation with their patients. This study aimed to explore the perspective of oncologists in Oman on fertility preservation. Methods: A cross-sectional study of physicians and surgeons dealing with patients with cancer was conducted from June 2021 to December 2021. A standardized and validated questionnaire was used to collect data. Results: Participants reported that they are knowledgeable about sperm cryopreservation and gonadotropin-releasing hormone agonists use but not other methods of fertility preservation. About 94% of the participants reported that they need more knowledge about fertility preservation. More than half of the participants had never encountered cancer patients who used ovarian cryopreservation, testicular tissue cryopreservation, in vitro fertilization with embryo cryopreservation and oocyte cryopreservation. The majority (78%) agreed that discussing fertility preservation with newly diagnosed cancer patients is a high priority. Conclusions: Oncologists in Oman are supportive of fertility preservation. The lack of knowledge and unavailability of well-structured fertility preservation services in Oman hinders the initiation of fertility preservation discussions.

## 1. Introduction

Exceptional achievement in the early diagnosis of cancer and its treatment causes an increase in life expectancy and a 5-year survival rate. Because of this phenomenal progress, there is a growing population of people who, while being cancer-free for several years, are concerned about the long-term impact of cancer or its treatments on their health, survival, and quality of life [1]. 

Chemotherapy and radiotherapy are the two most common cancer treatment modalities. Although these treatment modalities are playing a major role in the treatment of cancer, the side effects of chemotherapy and radiotherapy are unpreventable [2]. Premature mortality and long-term morbidity are possible long-term consequences of cancer and its treatment [3]. Non-target therapies such as chemotherapy and radiotherapy fail in the challenge of reaching full recovery with their receivers, as they highly affect dividing cells such as reproductive system cells. It affects the testicular tissue in males [4]. Moreover, it influences females’ fertility at different levels. It exerts influence on the ovarian reserve by leading to primordial follicle loss or damage [5]. This type of treatment also may cause central infertility for both males and females because it affects the hypothalamic–pituitary–gonadal axis [4]. Female survivors are facing a 40–80% risk of infertility. On the other hand, male survivors are facing a 30–70% risk [6]. Rates of cancer-related infertility in men and women depend on a number of factors including cancer itself, age, sex, diagnosis and treatment type and dose [7].

Given the growing number of survivors, it is time to coin the term “fertility preservation”, which refers to the maintenance of fertility in cancer patients after treatment. Fertility preservation options vary between male and female patients as well as between young and adult patients. Male fertility can be preserved by hormonal suppression, testicular sperm extraction, sperm cryopreservation, and testicular tissue preservation. Sperm cryopreservation is by far the simplest and commonest standard technique for post-pubertal males. Testicular tissue freezing and testicular sperm extraction are choices for pre-pubertal boys or men who are sperm deficient. In animal research, germ cell transplantation, germ cell maturation, and stem-cell-to-germ-cell progression have all shown potentials in preserving male fertility [8]. Females who wish to maintain their fertility may use the cryopreservation of oocytes, embryos, or ovarian tissue. Embryo cryopreservation is the most common and cost-effective form of fertility preservation nowadays. The cryopreservation of ovarian cells or oocytes may help female survivors become mothers in the future, even if they have been exposed to chemotherapy or certain infertility-causing agents [9,10]. The conjugating gonadotropin-releasing hormone given during the chemotherapy will reduce the damage to the female reproductive organs and oocytes during the treatment [11].

Loss of infertility has a devastating and long-lasting emotional impact on younger adults [12,13,14]. Furthermore, women, who do not have a discussion about their fertility with their health care professionals, have been reported to suffer higher levels of anxiety [15,16].

Counseling about cancer, its treatment, and fertility preservation should be given to all cancer patients, especially those who are at their reproductive age. Unfortunately, published works in this regard showed a lack of practice. Fertility preservation was not discussed with patients, and this topic was not included in the medical training programs [17]. Furthermore, several studies have shown that cancer patients in the United States are unaware of the detrimental effects of their cancer and its therapies on their fertility [18]. In addition, several studies in China show that patients are unaware of fertility preservation methods [17]. As oncologists are the first line of contact for patients newly diagnosed with cancer, gaining their insights on this topic is crucial to improve cancer survivors’ treatment and quality of life.

Many cancer survivors would prefer having their own biological child, and they are interested in preserving their fertility [19,20,21]. Early counseling about FP as soon as the diagnosis of cancer is being made is shown to lead to satisfactory results for such patients [15,19]. Many countries had issued their own regulation to the oncologists to map the good care that should be given to this group of patients [19]. However, to which extent these regulations are implemented in real practice is not well studied yet [19]. Many published research studies showed that the information received by the patients from their treating oncologists is perceived as inadequate if not given at an appropriate time [19,22,23]. Lack of a discussion with oncologists about the impact of cancer and its treatment on patients’ fertility is reported by more than one-third of patients in a study conducted in the United States [19]. Limitations hinder the initiation of such discussion, and providing the information needed to such patients is not well studied and needs to be explored.

Fewer studies are reported to investigate the fertility preservation in developing countries. A lack of awareness among clinicians, patients and caregivers was reported. Moreover, the insufficiency of financial support, absence of service and funding were reported as major barriers to practice fertility preservation in this part of the world. The engagement of stakeholders as well as collaboration and support from developed world countries were recommended [24]. In Oman, information related to the practices and views of physicians dealing with patients diagnosed and treated for cancer is limited. This is the first study that aimed to explore the oncologists’ knowledge, practice and attitudes toward fertility preservation in the Sultanate of Oman.

## 2. Materials and Methods

### 2.1. Study Setting

This study was conducted at Sultan Qaboos University in Oman from June 2021 to December 2021. This study involved oncologists, treating cancer patients and/or patients receiving chemotherapy and radiotherapy, from four different hospitals and centers.

### 2.2. Study Measures

The questionnaire used in this study was adapted from a published article by Adams et al. with minor changes [19]. Three experts modified the questionnaire and revised it for the context of practice in Oman before distribution. A pilot was conducted by fourteen oncologists filling in the questionnaire, and no changes were recommended. After validation, the questionnaire was used for data collection (Supplementary Materials Data S1).

The questionnaire is divided into two sections. The first section covers the participants’ demographic information, medical training and practice information. The second section investigated knowledge, current practice, barriers to fertility preservation, perceptions, and attitudes toward FP. 

### 2.3. Data Collection

The target participants of this study were all the oncologists dealing with cancer patients in four centers and hospitals treating patients with oncological diseases in Oman.

Information about the number of oncologists, their specialties, and their contact was obtained with the help of our collaborators in the hospitals and centers included in the study. The cohort of concern (oncologists dealing with cancer patients) includes 71 oncologists. Our sample size is calculated to be 61 participants with a 95% confidence level and a 5% margin of error. To increase the response rate, all 71 oncologists were invited to participate in the study via an email that included a detailed description of the project’s objectives and goals and contained a link to the questionnaire. Where feasible, face-to-face meetings were used in addition to the email invitations to increase the response rate. 

### 2.4. Ethical Approval

The Medical Research Ethics Committee, College of Medicine and Health Sciences, Sultan Qaboos University (REF.NO.SQU-EC/260/2020) approved this study. 

### 2.5. Statistical Analysis

The data analysis was performed using Statistical Package for the Social Sciences software (SPSS^®^). Descriptive statistics were used to present the demographic data and questionnaire items. In addition, the Chi-Square test was used to compare the categorical variables. For the unequal distribution of answers in some question, some items were turned into dichotomous items. For all statistical tests, a *p*-value < 0.05 was considered to be statistically significant. The qualitative comments analysis was abandoned, as the information provided did not add to that obtained by the survey.

## 3. Results

Thirty-four oncologists completed the questionnaire, giving a response rate of 48%.

About 68% of participants were male and 32% were female. More than half of the participants (56%) were older than 40 years old.

The majority (65%) of the oncologists that participated in the study were consultants, and about 62% of them reported that they have gone through personal experience of cancer and 94% reported that they have children (Table 1). 

### 3.1. Oncologists’ Knowledge about Fertility Preservation

**Figure 1:**
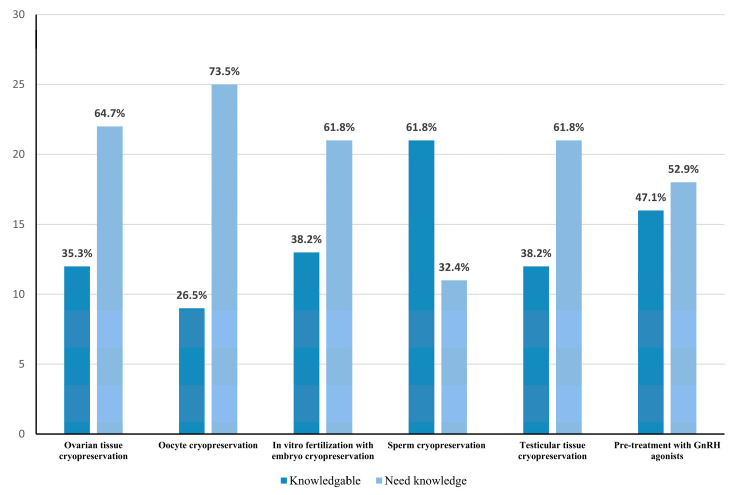
Oncologists’ knowledge about different fertility preservation methods.

Most of the participants reported that they were knowledgeable mainly about sperm cryopreservation (62%) and pretreatment with GnRH agonists (47%). On the other hand, few participants were aware about ovarian tissue cryopreservation (35.3%) and oocyte cryopreservation (27%) (Figure 1). About 94% of the participants reported that they needed to know more about FP. Among different methods of FP, the participants reported that they need more knowledge about oocyte cryopreservation (74%) and ovarian tissue cryopreservation (65%). There is no significant statistical association between the need for knowledge and the age or the current grade (the position on the carrier ladder) of the participants (*p*-value = 0.49 and 0.53, respectively).

### 3.2. Current Practice

Participants were asked about the frequency of encountering cancer patients who underwent different methods for FP. They reported that they encountered cancer patients who have used sperm cryopreservation (34%) and pretreatment with GnRH agonist (38%) most often. On the other hand, most physicians reported that they had never encountered a cancer patient who used ovarian tissue cryopreservation (66%), testicular tissue cryopreservation (65%), in vitro fertilization with embryo cryopreservation (59%), and oocyte cryopreservation (59%). 

About 71% of participants reported that they usually and/or always check with their patients the importance of their future fertility for them. Taking into consideration the desire of the patients for future fertility while planning patient’s treatment regimen is reported by 77% of the participants. Only four participants (12%) reported that they provided their patients with a written information about fertility preservation. The consultation of a fertility specialist or reproductive endocrinologist with questions about the patient’s fertility issues is reported by only four of the participants (12%). Nine of the participants (27%) reported always referring patients for fertility issues discussion with fertility specialists or reproductive endocrinologist prior to or after the cancer treatment. 

Participants reported that they discuss sperm cryopreservation (77%) and ovarian function suppression with LH-RH agonists (47%) more frequently compared to other methods of fertility preservation. 

Discussing and providing complete information about FP is one of the patient’s rights. There are many ways to provide cancer patients with enough information regarding the reservation of their fertility. About 68% of the participants believed that referring patients to a fertility specialist would significantly increase their knowledge of their fertility issues. Around 59% of the participants believed that providing educational documents about fertility would be beneficial for the patients to read. In addition, establishing a collaboration between hospitals and fertility centers to disseminate information and knowledge to the patient was reported to be helpful by 53% of the participants. Of the participants, 47% emphasized that continuing education of the patients is of great importance. Moreover, discussing the impact of cancer treatment on patients’ future fertility and providing patients with written information about FP are the participants’ preferred approaches to provide information about FP for those patients.

### 3.3. Attitudes of the Oncologists Towards Fertility Preservation

About 65% of the participants reported that they consider 40 as the upper age limit they would consider giving FP counseling and advice to a female patient. However, about 21% of them reported that they would consider providing FP advice to a male patient up to the age of 55. 

The majority of the participants seemed to be supportive and concerned about FP. The majority of them (78%) agree that discussing fertility preservation with newly diagnosed cancer patients is a high priority. However, 59% reported that treating the primary cancer is more important than FP. About 78% reported that they feel comfortable discussing fertility preservation with their patients. When they were asked about the FP success rate, 35% neither agree nor disagreed that the success rate of fertility preservation options is good enough to make it a viable option for their patients (Table 2). Oncologists were asked about the impact of gender, socioeconomic status and educational level of the patients on influencing patients’ attachments to their future fertility. The vast majority of the oncologists reported that these factors are influencing patients’ attachments to their future fertility. 

About 62% reported that they think gender is playing a role, and 27% of them thought that women are more concerned. Regarding socioeconomic states, 65% agreed that both high and low socioeconomic states patients are concerned about their future fertility, but 27% reported that the high socioeconomic state is more concerned. Lastly, regarding educational level, participants thought that patients of all educational levels are concerned about their future fertility.

### 3.4. The Main Barriers for Initiating Fertility Preservation Discussion

The majority of the participants (82%) reported that a lack of infrastructure of fertility services is a major barrier to initiate fertility preservation discussion with their patients. About 59% reported that their limited knowledge about fertility preservation options plays a major role. The priority to treat the cancer, cancer characteristics and prognosis are reported as barriers as well.

Patients’ desire to not discuss fertility preservation is reported by 88% of participants as a barrier hindering the initiation of FP discussion. Moreover, patient’s age is also playing a role here, as 74% of the participants reported that treating patients who aged more than 40 years might influence their initiation of FP discussion. Having emotionally distressed patients at the time of the consultation is reported to hinder the initiation of FP discussion by 71% of the participants. About 65% of the participants reported that treating a patient who has a child or children before influences their decision in initiating FP discussion (Table 3 and Table 4).

## 4. Discussion

Chemotherapy and radiotherapy for cancer patients negatively impact ovarian and testicular functions, especially those involving the whole body or mainly the pelvic region [25]. FP has gained increasing attention worldwide over the past decade due to the improvement in cancer treatment, leading to an increase in the number of cancer survivors. To the best of our knowledge, this is the first study to spot the level of awareness, knowledge, current practice, attitudes and barriers toward fertility preservation in Oman. This study can be most closely compared with a previous survey of oncologists’ current knowledge, practice and attitudes in the United States of America (USA) by Adam [19], and another study from the United Kingdom (UK) by Forman et al. [26] as well as a study from Arabic communities by Rabah et al. [27].

Nearly half, 48%, of the participants responded to the questionnaire, indicating a substantial interest from some oncologists in FP. An important result that emerged from the data of this study is that a vast majority (94%) of our participants indicated that they need more knowledge about FP. This was a very high percentage compared to a study conducted in Japan in which 36% of participants expressed the same [28]. Multiple studies on FP knowledge were conducted worldwide, and a common conclusion amongst all is that there is a pressing need to create better education campaigns to increase awareness about the various methods of FP [19,28,29]. A common argument put forth regarding the level of insufficient knowledge regarding FP is that it is due to the lack of common guidelines that oncologists and physicians can refer to.

Regarding the knowledge about different fertility preservation methods, the most knowledgeable fertility preservation methods were sperm cryopreservation 68% and pretreatment with GnRH agonists 47%. Furthermore, this has a complete agreement with previous studies [19,25]. Despite this, sperm cryopreservation is a simple and effective way to preserve fertility in male patients who only need to provide a semen sample for cryopreservation prior to chemotherapy [25]. This may be because it is the most successful method practiced to preserve fertility worldwide [30]. Regarding pretreatment with GnRH agonists, it has been a long time since this method was used in medical applications with proven safety and efficacy, and this may explain the fact that most of the participants are knowledgeable about this method [11,31,32]. In this study, no association was found between the need for knowledge and the participants’ age or grade. Similar findings were reported by Adams et al. [19].

Most of the participants reported that they encountered patients who had used pretreatment with GnRH agonists 38% and sperm cryopreservation 34% most often. Furthermore, this is not surprising, because they reported before that they were knowledgeable mainly in those two methods. Moreover, when recalling the history of FP, the first live birth using sperm cryopreservation was reported in 1953 compared to ovarian tissue cryopreservation, which is a recent milestone in the field [33]. According to our findings, most of our participants knew little about ovarian tissue and oocyte cryopreservation (35.3%, 27%, respectively). Moreover, this fits well with Adams et al. [19]. Parallel to this, a large number of our participants reported that they had never encountered patients who underwent ovarian tissue and oocyte cryopreservation (66% and 59%, respectively). This result is consistent with published findings [19].

Our study showed that oncologists seemed supportive of FP, with 78% agreeing or strongly agreeing that FP is a high priority to discuss with their newly diagnosed cancer patients. These findings go hand in hand with what is reported in Adam et al. Moreover, 78% of the oncologists reported that they feel comfortable discussing fertility preservation with their patients, which is a higher percentage than what was reported in Adams et al. (65%). However, 59% also agreed that treating the primary cancer was more important than FP, which is comparable to what had been reported in Adams et al. that 67% of their participants agreed with same statement. Owing this inconsistency to one of the barriers, which is the limited knowledge about fertility preservation options. Given that the majority of participants believed that treating the main cancer is more crucial than preserving fertility, it is hoped that the clinician’s understandable attitude does not impede patients from obtaining the information they need to make an informed decision. For the success rate of fertility preservation methods, 53% agreed or strongly agreed with the statement “success rates of FP are good enough to make it a viable option”. This is comparable to what had been reported in Adam et al. [19]. However, the percentage reported by Adam et al. (82%) is higher than what we reported, and this can be explained by the advanced program of cancer care in other parts of the world where a FP program is included in oncology care. However, the percentage we reported here made us optimistic that the oncologists participated in this study are reading about the results of fertility preservation options. Having said that, these results should be taken and analyzed with caution, as it is obtained from a self-administrated questionnaire which may not reflect the actual practice or attitude.

It is imperative to provide accurate and up-to-date information, particularly on a subject as complex as FP, for those patients, which would help them make more informed choices regarding their fertility. Genetic counseling should be included when providing fertility preservation counseling to patients with hereditary cancer syndrome due to their peculiar needs. Personalized counseling that explains the risk of transmission of their genetic cancer susceptibility to their offspring is crucial. There are different ways to provide cancer patients with enough knowledge about FP. Most of our participants preferred referring their patients to a fertility specialist, 68%. When a patient is diagnosed with cancer, and before treatment begins, early referral to a fertility specialist is vital for maximizing the chances of fertility preservation [17]. This was well demonstrated in the study by Adams et al., in which 67% of their participants reported that they referred their patients to a fertility specialist [19]. On the other hand, a study by Rabah et al. showed that less than 20% of their participants reported that they regularly referred their patients to a fertility specialist [27].

Concerning information provision, more than half (59%) of the participants believe that providing educational documents about fertility for the patients to read would be beneficial. However, many factors may explain why some physicians do not provide their patients with written documents. We assume that the hospitals’ lack of structured written information for their physicians will impact their ability to provide written information. Another aspect is that due to their lack of understanding of FP, physicians will supply any information they can regarding FP to their patients, even if the information is inaccurate or not up to date. According to another survey, about 70% of physicians believe that ensuring prolonged patient survival is more essential than FP [32]. As a result, they will not provide information on FP to the patients, as they believe that it is not of utmost priority. In addition, around 53% of our participants support the idea that finding a collaboration between hospitals and fertility centers will enhance transferring information for cancer patients. Nevertheless, lacking a specific program or center for fertility preservation in Oman resulted in a lack of resources for written information to the patients.

Having a higher percentage of oncologists who thought that women were more concerned than men about preserving their fertility can be explained by the woman’s ovaries containing a finite number of primordial follicles (termed ovarian reserve) that declines over time and at a higher rate when exposed to chemotherapy. A higher percentage of oncologists thought that patients with high socioeconomic status were more concerned than those with low socioeconomic about preserving their fertility. Few studies investigating the impact of socioeconomic status on FP showed similar results, as FP options are expensive, and high socioeconomic status patients could afford it [34,35]. Regarding educational level, there is no difference reported by the oncologists between all levels. Many studies showed the same results as we reported in our study [19,26,36].

As seen from oncologists’ responses, the existing infrastructure of fertility services seemed to be perceived as a major barrier to providing FP by participants. These findings are in contrast to Adams et al. and Forman et al., who found that fertility services did not seem to be perceived as a major barrier, as the vast majority of participants in their studies reported FP options were available within their trust [19,26]. This is an indication that there is a lack of fertility services in Arabic communities [27]. Such a high percentage indicates that the lack of fertility preservation services in Oman is a significant barrier that needs to be investigated and worked on by building the infrastructure required and providing these services to cancer patients.

The vast majority of our participants reported that the priority to treat the cancer is influencing them to some extent or a large extent. In contrast, in many published studies, it is found that the discussion of FP has medium to low priority for the oncologists. About half of the oncologists reported their own lack of knowledge about FP as a determining factor. That is agreed with what is reported by Adams et al. [19]. 

Given the dearth of knowledge reported by the majority of oncologists, it may come as a surprise that the majority claimed they were comfortable addressing FP with patients and that it was a priority. However, addressing the impact of cancer therapy on fertility does not necessitate a thorough understanding of fertility; rather, it is probably more important to evaluate whether FP is appropriate for the woman or couple in question and to know where to refer. Nonetheless, the findings raise the question of what frequent fertility chats might entail and what information might be provided. It would also be fascinating to study how patients interpret this information flow, and additional research is needed to gain a better understanding of their viewpoints. 

The vast majority stated that cancer prognosis plays a major role. Forman et al. agreed with that, as 88% of their respondents reported that their decision would be influenced by the patients’ poor prognosis [26]. However, only 30% of Adams et al. respondents agreed with the same statement [19]. We attributed this big difference to the fact that oncologists who participated in Adams et al. [19] were aware of different FP options.

About 88% of the participants stated that having a patient who does not want to discuss fertility preservation is a determining factor for FP discussion. A study published by King et al. participants stated that they would not talk about FP options unless the patients show that they are interested [37]. The vast majority of oncologists stated emotional distress at the time of consultation plays a major role. The window in which a decision about FP can be made is frequently limited, and it occurs during the chaotic and emotionally charged period that follows a cancer diagnosis. Involving a psychologist in the discussion has the potential of improving this situation. More than half of the participants stated that having a patient who already has a child or children and is not concerned about future fertility is influencing their decision to start a discussion of FP with their patients. That is agreed by Adams et al., as 44% of participants stated that whether the patient already had children may influence their decision to discuss FP [19]. This could be considered as the main difference between our findings and those of Forman et al., as they reported that only 10% of the respondents said that having a patient who has children would influence their decision to discuss FP [26].

## 5. Conclusions

This study presents for the first time the awareness level of oncologists in Oman about fertility preservation for patients with cancer. Overall, the study presented an encouraging picture, with the majority of oncologists seeming supportive of FP and feeling comfortable discussing fertility preservation with their patients. However, the study showed that there is a lack of knowledge and a lack of fertility service in Oman, which may be considered major obstacles preventing oncologists from discussing fertility preservation with their patients. The overall lack of knowledge of FP methods suggests that oncologists may benefit from further education and information about current reproductive medicine procedures available.

Providing cancer patients with needed information related to their fertility is vital. Therefore, collaboration among different gulf region countries or Arab countries and other parts of the world with advanced care in this regard is needed to formulate a standard guideline for physicians when dealing with cancer patients, including fertility preservation. 

## Figures and Tables

**Table 1:** General characteristics of participants.

Characteristics	Number of Participants	%
Sex		data
Male	23	67.6
Female	11	32.4
Age		
≤40	15	44.1
>40	19	55.9
Current grade		
Consultant	22	64.7
Other than consultant	12	35.3
Having children		
Yes	32	94.1
No	2	5.9
Personal experience of cancer		
Yes	21	61.8
No	13	38.2

**Table 2:** Attitudes of the oncologists toward fertility preservation.

	Strongly Disagree	Disagree	Neutral	Agree	Strongly Agree
Fertility preservation is a high priority for me to discuss with newly diagnosed cancer patients	3.1%	0%	18.8%	37.5%	40.6%
Treating the primary cancer is more important than fertility preservation	3.1%	21.9%	15.6%	40.6%	18.8%
The success rate of fertility preservation are not as yet good enough to make it a viable option	15.6%	37.5%	37.5%	6.3%	3.1%
I feel comfortable discussing fertility preservation with my patients	3.1%	3.1%	15.6%	53.1%	25%

**Table 3:** Medical factors as barriers for initiating fertility preservation discussion with oncology patients.

To What Extent Would You Say the Following Medical Factors Influence Whether or Not You Initiate a Discussion about Fertility with a Patient?	Not at All	To Some Extent	To Large Extent
Lack of fertility service in the area	17.6%	53%	29.4%
Constraints on my time	64.8%	29.4%	5.8%
My limited knowledge about fertility preservation options	41.2%	55.9%	2.9%
The priority to treat the cancer			
Someone else within my practice discuss fertility preservation with my patients	26.5%	38.2%	14.7%
Patient is too ill to delay treatment to pursue fertility preservation	17.6%	53%	29.4%
Fertility treatment may present a risk for cancer patients	50%	41.2%	8.8%

**Table 4:** Patients’ related factors as barriers for initiating fertility preservation discussion with oncology patients.

To What Extent Would You Say the Following Factors Influence Whether or Not You Initiate a Discussion About Fertility with a Patient?	Not at All	To Some Extent	To Large Extent
Patient is single	47.1%	35.3%	17.6%
Already has a child or children and not concerned about future fertility	29.4%	38.2%	26.4%
Patients does not want to discuss fertility preservation	11.8%	76.4%	11.8%
Age more than 40 years	26.5%	55.9%	17.6%
Age less than 18 years	47.1%	38.2%	14.7%
Emotional distress at the time of consultation	29.4%	55.9%	14.7%

## Data Availability

Data is unavailable due to ethical restrictions.

## Supplementary Materials

The following supporting information can be downloaded at: https://www.mdpi.com/article/10.3390/life13030801/s1, Data S1: The main questionnaire.
