# Supplementary material for: Oncologists’ Knowledge, Practice and Attitude toward Fertility Preservation: A National Survey
Source: Life (Basel). 2023 Mar 15;13(3):801. doi: 10.3390/life13030801 (PMC10054663; doi:10.3390/life13030801)
Supplement: Supplementary file 1 [file life-13-00801-s001.zip › life-2233505-supplementary.pdf]

Supplementary Data 1: The main questionnaire

Personal information

Gender

- o Male
- o Female

Age

- o Under 30
- o 31–40
- o 41–50
- o 51–60
- o 61 or over

Do you have children?

- o Yes
- o No

Have you or a close family member had cancer?

- o Yes
- o No

What is your specialty?

- o Medical/clinical oncology
- o Surgical oncology
- o Other oncology, please specify

In which cancer subspecialty you are specialized?

- o Breast
- o Gynaecological
- o Urological
- o Gastrointestinal
- o Lung
- o Head and neck
- o Haematological
- o CNS
- o Paediatric
- o Sarcomas/soft tissue
- o Other: please specify

What grade are you currently?

- Consultant
- Specialist

How many cancer patients do you usually have in your care in each of the following age ranges? (check all that apply)

	Less than 30%	30-60%	More than 60%
0-18 years			
19-45 years			
46 years and over			

Approximately, what percentage of your oncology patients aged 0-18 years is: (check all that apply)

	Less than 30%	30-60%	More than 60%	None
Pre-pubertal				
Pubertal				

Approximately, what percentage of your oncology patients aged from 18 to 45 years is

	Less than 30%	30-60%	More than 60%	None
Men				
Women				

Estimate the percentage of each type of cancer in your regular activity. (Check the appropriate boxes):

	Less than 30%	30-60%	More than 60%
Breast cancer			
Cervical cancer			
Other gynecological cancers			
Blood cancers			
Colorectal cancer			
Other cancers			

If other, please specify.

How would you describe your level of knowledge of the following fertility preservation options?

	Not aware, need more knowledge	Aware of, but need more knowledge	knowledgeable	Very knowledgeable
Ovarian tissue cryopreservation				
Oocyte cryopreservation				
In vitro fertilization with embryo cryopreservation				
Sperm cryopreservation				
Testicular tissue cryopreservation				
Pre-treatment with GnRH agonists				

Do you feel you need more knowledge about fertility preservation options?

- o Yes
- o No

Which of the following best represents the age up to which you would consider fertility preservation advice relevant for a woman? Check all that apply.

- o Less than 20
- o 20-30
- o 31-40
- o 41-45
- o 46-50
- o 51-55
- o 56-60

Which of the following best represents the age up to which you would consider fertility preservation advice relevant for a man?

- o Less than 20
- o 20-30
- o 31-40
- o 41-45
- o 46-50
- o 51-55
- o 56-60

Have you encountered patients who have had/are having fertility preservation?

- o Yes
- o No

If YES, how often do you encounter patients who have used/are using one of the following fertility preservation options?

	Never	Rarely	Sometimes	Often
Ovarian tissue cryopreservation				
Oocyte cryopreservation				
In vitro fertilization with embryo cryopreservation				
Sperm cryopreservation				
Testicular tissue cryopreservation				
Pre-treatment with GnRH agonists				

How often do you do each of the following with patients of child-bearing age?

	Never	Rarely	Usually	Always
I check with the patient how important their future fertility is for them				
When I plan the patient`s treatment regimen I take into account their desire for future fertility				
I discuss the impact of the patient`s condition and/or treatment might have on their future fertility				
I provide my patients with written information about fertility preservation				
I consult fertility specialist or reproductive endocrinologist with questions about potential fertility issues in my patients				
I refer patients who have questions about fertility to a fertility specialist or reproductive endocrinologist prior to starting the treatment (chemotherapy and radiotherapy)				
I refer patients who are concerned about their fertility to fertility specialist or reproductive endocrinologist after the treatment (chemotherapy and radiotherapy)				

Approximately how many of your patients have been referred to a fertility specialist and/or gone on to have fertility treatment in relation to their cancer treatment in the last year?

	0	1-5	6-10	More than 10
Referral				
Treatment				

For patients who wish to preserve their fertility before undergoing cancer treatment:
(check which corresponds to your usual practice, check all that apply)

- I refer my patients to a dedicated consultation in my oncology department/center
- I refer my patients to a referent fertility center for this type of treatment
- I provide my patients with a list of referent fertility centers
- I ask my patients to contact a gynecologist
- I refer my patients to another professional (practitioner or association)
- None of the above

Which methods of fertility preservation do you discuss with your patients?
(check all that apply)

- Oocyte cryopreservation
- Ovarian tissue cryopreservation
- Ovarian transposition
- Embryo cryopreservation
- Ovarian function suppression with LH-RH agonists
- I don't mention specific methods
- I don't discuss fertility preservation with my patients

What might help you to provide your patients with information on fertility preservation? (check all that apply)

- Documentation (brochures…) to give to patients
- Continuing education
- A fertility specialist who consults in my department/center
- A collaboration with a fertility center (network)
- None of the above

How much do you agree or disagree with each of the following statements?

	Strongly disagree	disagree	Neither agree nor disagree	agree	Strongly agree
Fertility preservation is a high priority for me to discuss with newly diagnosed cancer patients					
Treating the primary cancer is more important than fertility preservation					
The success rate of fertility preservation are not as yet good enough to make it a viable option					
I feel comfortable discussing fertility preservation with my patients					

To what extent do you think that the following are factors in the importance patients attach to their future fertility?

	Yes	no
Gender		
Socioeconomic status		
Educational level		
Cultural background		

Whom do you think is more concerned about preserving fertility?

- Men
- Women
- Both

Whom do you think is more concerned about preserving fertility?

- Patient with higher socio-economic status
- Patient with lower socio-economic status
- Both

Patients from which educational level in your experience are most concerned about future fertility?

- School
- University
- Postgraduate qualification
- All levels

Please choose the best option to each statement in the following:

	Not at all	To some extent	To large extent
**To what extent would you say the following medical factors influence whether or not you initiate a discussion about fertility with a patient?**			
Poor success rate of fertility preservation options			
Lack of fertility service in the area			
Constraints on my time			
My limited knowledge about fertility preservation options			
The priority to treat the cancer			
Someone else with my practice discusses fertility preservation with my patients			
Is too ill to delay treatment to pursue fertility preservation			
Fertility treatment may present a risk for cancer patients			
Aggressive cancer requiring urgent treatment			
A history of cancer may contraindicate pregnancy			
Has a hormonally sensitive malignancy			
Patient has a poor prognosis			
**To what extent would you say the following medical factors influence whether or not you initiate a discussion about fertility with a patient?**			

Patient is single			
Already has a child or children and not concerned about future fertility			
Patient does not want to discuss fertility preservation			
Age more than 40 years			
Patient is minor (less than 18)			
Emotional distress at the time of consultation			
Other			

If other, please specify.

Please add any other comments you may have regarding fertility preservation and your patients
